# Segmentation of the Cingulum Bundle in the Human Brain: A New Perspective Based on DSI Tractography and Fiber Dissection Study

**DOI:** 10.3389/fnana.2016.00084

**Published:** 2016-09-07

**Authors:** Yupeng Wu, Dandan Sun, Yong Wang, Yibao Wang, Shaowu Ou

**Affiliations:** ^1^Department of Neurosurgery, The First Affiliated Hospital of China Medical UniversityShenyang, China; ^2^Department of Cardiovascular Ultrasound, The First Affiliated Hospital of China Medical UniversityShenyang, China

**Keywords:** cingulum bundle, white matter, diffusion spectrum imaging, fiber dissection, Klingler’s technique

## Abstract

The cingulum bundle (CB) is a critical white matter fiber tract in the brain, which forms connections between the frontal lobe, parietal lobe and temporal lobe. In non-human primates, the CB is actually divided into distinct subcomponents on the basis of corticocortical connections. However, at present, no study has verified similar distinct subdivisions in the human brain. In this study, we reconstructed these distinct subdivisions in the human brain, and determined their exact cortical connections using high definition fiber tracking (HDFT) technique on 10 healthy adults and a 488-subject template from the Human Connectome Project (HCP-488). Fiber dissections were performed to verify tractography results. Five CB segments were identified. CB-I ran from the subrostral areas to the precuneus and splenium, encircling the corpus callosum (CC). CB-II arched around the splenium and extended anteriorly above the CC to the medial aspect of the superior frontal gyrus (SFG). CB-III connected the superior parietal lobule (SPL) and precuneus with the medial aspect of the SFG. CB-IV was a relatively minor subcomponent from the SPL and precuneus to the frontal region. CB-V, the para-hippocampal cingulum, stemmed from the medial temporal lobe and fanned out to the occipital lobes. Our findings not only provide a more accurate and detailed description on the associated architecture of the subcomponents within the CB, but also offer new insights into the functional role of the CB in the human brain.

## Introduction

In humans, the cingulate cortex forms an arch, extending from the rostral subcallosal area anteriorly and following the curved superior surface of the corpus callosum (CC) bilaterally on the sagittal plane (Haznedar et al., 2004). The cingulum bundle (CB) is a 5–7 mm in diameter fiber bundle that locates above the CC and under the cingulate cortex (Schmahmann and Pandya, 2006). It originates within the white matter of the temporal pole, runs posterior and superior into the parietal lobe, then turns, forming a “ring-like belt” around the CC, into the frontal lobe, terminates anterior and inferior to the genu of the CC in the orbital-frontal cortex (Agrawal et al., 2011). Numerous studies have demonstrated that the CB is involved in core processes such as executive function, decision-making, and emotion processing (Heilbronner and Haber, 2014; Adnan et al., 2016).

In 1995, based on cytoarchitectural and receptor architecture work, Vogt et al. (1995) suggested that the cingulate cortex might be subdivided into the anterior, mid, posterior and retrosplenial cortices (ACC, MCC, PCC and RSC, respectively). The ACC is characterized by neurons expressing high levels of dopamine D1 receptors, projecting to brainstem motor systems, and which is believed to be predominantly involved in emotion and autonomic regulation (Benes, 1998; Haznedar et al., 2004). The MCC has neurons projecting to the spinal cord, which play an important role in emotional processing and visceral control (Vogt, 2016). The PCC contains large numbers of neurofilament protein-containing neurons, connecting with the parietal lobe, that monitor eye movements and respond to sensory stimuli (Vogt et al., 1992). The RSC is mainly formed by neurons receiving input from the amygdala and is known to be involved in cognitive function (Vogt and Laureys, 2005). Thus, the traditional segmentation of the CB into anterior, middle, and posterior portions (Concha et al., 2005; Jang et al., 2013), has been widely suggested by studies of functional imaging, electrical stimulation, as well as some patient studies. The anterior portion is predominantly known to be involved in executive function, decision-making, and emotion (Catheline et al., 2010; Tuladhar et al., 2015). The middle portion participates in the execution of motor- and attention-related tasks (Lin et al., 2014). The posterior region is related to cognitive function (Choo et al., 2010; Sexton et al., 2011).

In the last decade, with the rapid development of the diffusion tensor imaging (DTI) technique, we have the opportunity to study the CB *in vivo*. Based on DTI tractography, some studies have revealed that the CB is a complex network structure, containing not only long fibers that potentially extend to the frontal and temporal lobes, but also short association fibers connecting adjacent cortices (Catani and Thiebaut de Schotten, 2008; Lawes et al., 2008). According to the segmentation in the anterior-middle-posterior direction, the CB resembles an uninterrupted and continuous band, regardless the presence of numerous short association fibers. Moreover, the traditional segmentations, noted above, do not adequately explain how the functional subcomponents of the CB system connect to the different cortical and subcortical regions, which performs as a “multi-task bundle” (Jones et al., 2013; Heilbronner and Haber, 2014; Sethi et al., 2015).

Although DTI tractography has provided us with a new version of the CB, there are several technical limitations, for instance, inability to map fiber endings of the CB before contacting the cortical mantle, failure to solve fiber crossings and to follow bundles within the CB, and excessive false fiber continuity generating pseudotracts (Fernandez-Miranda, 2013; Fernández-Miranda et al., 2015). Fortunately, all these DTI problems above can be addressed by some emerging fiber mapping techniques such as high-angular-resolution diffusion imaging (HARDI) and diffusion spectrum imaging (DSI). HARDI has better angular resolution and smaller voxels (Jansons and Alexander, 2003), while DSI is essentially a model-free imaging approach that has the ability to map complex fiber architecture at the scale of single MRI voxels (Wedeen et al., 2005). Recently, a novel combination of processing, reconstruction, and tractography methods called high definition fiber tracking (HDFT) has been developed (Fernandez-Miranda et al., 2012). It employs DSI reconstructed by generalized q-sampling imaging (GQI) as a high angular resolution based approach (DSI for acquisition, GQI for estimation of fiber orientation) that leverages high directional sampling of diffusion imaging space to get better resolution of underlying white matter geometry for tractography (Yeh et al., 2010). Studies have been shown that the HDFT provides accurate replication of complex known neuroanatomical features where DTI failed (Wang et al., 2013; Fernández-Miranda et al., 2015; Wu et al., 2016).

Traditionally, experimental observations in non-human primates that employ axonal tracing are considered as the “gold standard” for guiding human white matter connective patterns (Schmahmann and Pandya, 2006; Thiebaut de Schotten et al., 2012). Mufson and Pandya (1984) found three distinct fiber components of the CB in the rhesus monkey using an autoradiographic tracer technique. Subsequently, experiments in monkeys revealed both the afferent and efferent fibers that occupied specific parts in the CB system, connecting various cortical and subcortical regions (Vogt and Pandya, 1987; Kobayashi and Amaral, 2007). Evidence has accumulated suggesting that the human CB may exhibit a similar segmentation regarding corticocortical connections. Jones et al. (2013) tested the validity of dividing the CB into three subdivisions corresponding to the “para-hippocampal”, “retrosplenial”, and “subgenual” portions based on DTI tractography, which provided a rough framework for the possibility of subdividing the CB, although further confirmation was necessary. In the living humans, the autoradiography tract tracing is inapplicable. Although it is not possible to use autoradiographical tracing in living humans, fiber dissection in post-mortem brains has been widely employed to cross-validate *in vivo* fiber tracking results. To date, however, there have been no studies employing such strategies.

In this study, we mapped the CB in the human using HDFT. Tractography of the CB were performed using both a subject-specific approach (10 subjects) and a template approach (Human Connectome Project, HCP-488). Complemented anatomical fiber dissections were also carried out to confirm the *in vivo* fiber tracking findings. Our study provided a more accurate and detailed description on the associated architecture of the subcomponents within the CB, contributing a comprehensive morphological basis for further functional studies.

## Materials and Methods

### Participants

Ten neurologically healthy volunteers (3 males, 7 females; all right handed; age range: 20–40 years; mean age, 33.5 years) participated in the experiment between September 2013 and October 2014 at the First Affiliated Hospital of China Medical University. All participants were prescreened prior to scanning to rule out any contraindications to MRI. This study was approved by the Ethics Committee of the First Affiliated Hospital of China Medical University (permit number AF-SOP-07-1, 0-01), and carried out in accordance with the Declaration of Helsinki. Written informed consent was obtained from each participant.

Except for subject-specific analysis, we also conducted fiber tracking on a template of 488 subjects from the HCP-488, which represented the largest and highest quality data available to date. The HCP consortium led by Washington University, University of Minnesota, and Oxford University, is undertaking a systematic effort to map macroscopic human brain circuits and their relationship to behavior in a large population of healthy adults. A total of 500 subjects’ data was released for the first three quarters (Q1–Q3, June 2014) and 488 subjects had diffusion scans (289 females, 199 males, average age 29.15, SD ± 3.47; Van Essen et al., 2013). The reconstructed data of the 488 subjects were averaged to create a representative template (HCP 488-subjects template is freely downloadable at: http://dsi-studio.labsolver.org/download-images).

### Image Acquisition and Reconstruction

We used a 3-T Tim Trio System (Siemens) with a 32-channel head coil to acquire DSI data. This involved a 43-min, 257-direction scan using a twice-refocused spin-echo echo-planar imaging sequence and multiple *q* values (repetition time (TR) = 9.916 ms, echo time (TE) = 157 ms, voxel size = 2.4 × 2.4 × 2.4 mm, field of view (FoV) = 231 × 231 mm, *b*_max_ = 7000 s/mm^2^; Wedeen et al., 2005, 2008). We also included the high-resolution anatomical imaging to be the anatomical comparisons, employing a 9-min T_1_-weighted axial magnetization prepared rapid gradient echo (MPRAGE) sequence (TR = 2110 ms, TE = 2.63 ms, flip angle = 8°, 176 slices, FoV = 256 × 256 mm^2^, voxel size = 0.5 × 0.5 × 1.0 mm^3^; Wu et al., 2016). DSI data were reconstructed with a GQI approach (Yeh et al., 2010). The orientation distribution functions (ODFs) were reconstructed to 362 discrete sampling directions and a mean diffusion distance of 1.2 mm. ODF is a probabilistic density function on the two-dimensional (2D) surface of a sphere, that can be calculated from diffusion MRI signals. The local maximums in an ODF are often regarded as the axonal fiber orientations in deterministic fiber tracking, a method that calculates the axonal trajectories between cortical areas. DSI is a diffusion MRI q-space imaging method. It acquires diffusion signals with multiple *b*-values to calculate average propagator and the diffusion ODF. The diffusion encoding sampling scheme of DSI is arranged as grid points in the q-space and distinguishes itself from the common-used shell arrangement which is limited to single diffusion gradient strength. The multiple diffusion gradient strength design gives DSI better coverage on both fast and slow diffusion (Yeh et al., 2013).

### The Human Connectome Project 488-Subject Template (HCP-488)

The 488 subjects underwent diffusion scans in a Siemens 3T Skyra scanner using a 2D spin-echo single-shot multiband EPI sequence with a multi-band factor of three and monopolar gradient pulse. The spatial resolution was 1.25 mm isotropic, TR = 5500 ms, TE = 89 ms. A multishell diffusion scheme was used. The *b*-values were 1000, 2000 and 3000 s/mm^2^. The total number of diffusion sampling directions was 270. The total scanning time was around 55 min (Sotiropoulos et al., 2013; Van Essen et al., 2013). The diffusion data were conducted using a GQI approach.

### White Matter Fiber Tracking

For the fiber-tracking datasets, all fiber tracking was performed using DSI Studio^1^. A whole brain seeding approach using multiple or a single region of interest (ROI) and region of avoidance (ROA) masks was performed. In voxels with multiple fiber orientations, fiber tracking was initiated separately for each orientation, and fiber progression continued with a step size of 1.2 mm, minimum fiber length of 20 mm, and turning angle threshold of 60° (Wang et al., 2013). If multiple fiber orientations existed in the current progression location, the fiber orientation that was nearest to the incoming direction and forms a turning angle smaller than 60° was selected to determine the next moving direction. To smooth each track, the next moving directional estimate of each voxel was weighted by 20% of the previous incoming direction and 80% of the nearest fiber orientation (Fernández-Miranda et al., 2015). This progression was repeated until the quantitative anisotropy (QA) of the fiber orientation dropped below a preset threshold (0.03–0.06 depending on the subject) or there was no fiber selected within the 60° angular range in the progression (Yeh et al., 2013). Once tracked, all streamlines were saved in the TrackVis file format. Segmentation of the CB was performed using DSI studio software. For comparison, FreeSurfer was used to segment cortical gyral ROIs using each participant’s T1-weighted MPRAGE image (Desikan et al., 2006).

**Figure 1:**
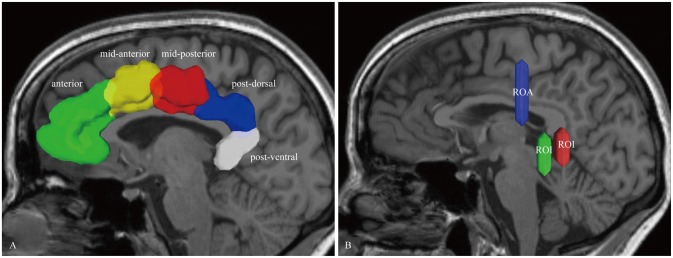
***In vivo* fiber tractography of the cingulum bundle (CB). (A)** The region of interests (ROIs) of the CB consist of five segments: anterior (green), mid-anterior (yellow color), mid-posterior (red color), post-dorsal (blue color) and post-ventral (white color) cingulate cortex. **(B)** Two ROIs and one region of avoidance (ROA) are used for reconstructing the para-hippocampal CB. The ROIs are placed behind and below the splenium. One ROA is placed above the body of the corpus callosum (CC). CB, cingulum bundle; ROI, region-of-interest; ROA, region of avoidance.

For the dorsal CB reconstruction, the ROI masks included the anterior, mid-anterior, mid-posterior, post-dorsal and post-ventral cingulate cortex (defined by the Automated Anatomical Labeling (Desikan et al., 2006); Figure 1A). If one label was selected to serve as seed region for the fiber-tracking algorithm, its neighboring mask was chosen for the subsequent ROI. Thus, this step repeated four times. Then, for the ventral CB, we used the methods described by Jones et al. (2013) in brief, two ROIs were placed behind and below the splenium. One ROA was placed above the body of the CC (Figure 1B).

### Fiber Dissection Technique

Eight hemispheres from four fresh specimens (age 38–77 years, 2 females, 2 males) were obtained from the Department of Anatomy of the First Affiliated Hospital of China Medical University. All the legal representatives signed the informed consent. The operations were approved by the Ethics Committee of the First Affiliated Hospital of China Medical University (permit number AF-SOP-07-1, 0-01). Postmortem fiber dissection was performed according to the technique originally described by Professor Josef Klingler (Ludwig and Klingler, 1965; Agrawal et al., 2011). The specimens were fixed in 10% formalin solution for four additional weeks, then frozen at −15°C for 15 days after removal of vessels, arachnoid and pia-matter. The water crystallization induced by the freezing process disrupts the structure of the gray matter (with high water content), enabling us to peel off the cortex from the brain surface. The freezing process also spreads along the white matter fibers, facilitating the dissection of the fiber tracts (Martino et al., 2011). Subsequently, the specimens were washed under running water for several hours.

We performed the dissection of each specimen before the anatomy of the lateral, medial and the basal cerebral surface was carefully studied. Then, the white matter dissection was completed in a stepwise manner for the medial-to-lateral dissection process. Our dissection tools were handmade, thin, and wooden dissectors, various sized anatomic forceps. Digital images were acquired during the course of dissection. The first step of dissection was removing the cortex within the depth of the sulci, with preservation of the cortex of the hemispheres surface of the gyri. Then the short associational U shaped fibers (also known as intergyral or arcuate fibers), which interconnected neighboring gyri at the subgyral sector, were exposed. After cutting off the U fibers, the deep CB white matter was exposed. The Atlas of the fiber pathways of the brain (Schmahmann and Pandya, 2006) was used to identify and define the specific cortical connections.

We undertook the fiber dissection studies at the Surgical Neuroanatomy Lab of the China Medical University with the aid of microsurgical instrumentation and surgical microscope (6–40 magnification, Carl Zeiss, OPMI CS-NC).

## Results

### Trajectory of the CB in the Human Brain

#### Subject-Specific Tractography Findings

**Figure 2:**
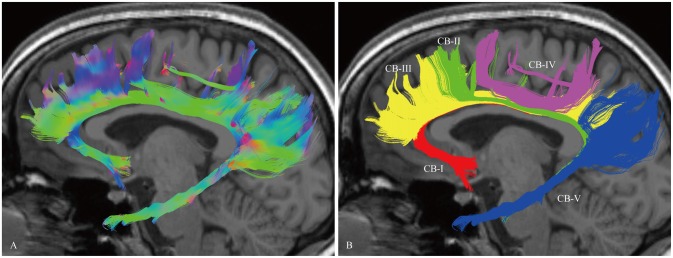
***In vivo* fiber tractography of the CB and five potential subcomponents in the CB (subject-8, left). (A)** Sagittal view of the left CB. The CB exhibits a sickle-shaped association bundle that nearly encircled the CC and extends to the temporal lobe. **(B)** Defining the subgenual areas, medial aspect of the superior frontal gyrus (SFG), precuneus, splenium and para-hippocampal gyrus as the cortex landmarks to do back-tracing for the subdivision (defined by the Automated Anatomical Labeling (Desikan et al., 2006)). Five subcomponents are identified (CB-I, red color; CB-II, green color; CB-III, yellow color; CB-IV, purple color; CB-V, blue color).

In the fiber tractography study, the CB exhibited a sickle-shaped association bundle that nearly encircled the CC and extended to the temporal lobe. Five potential subcomponents were identified (Figure 2).

**Figure 3:**
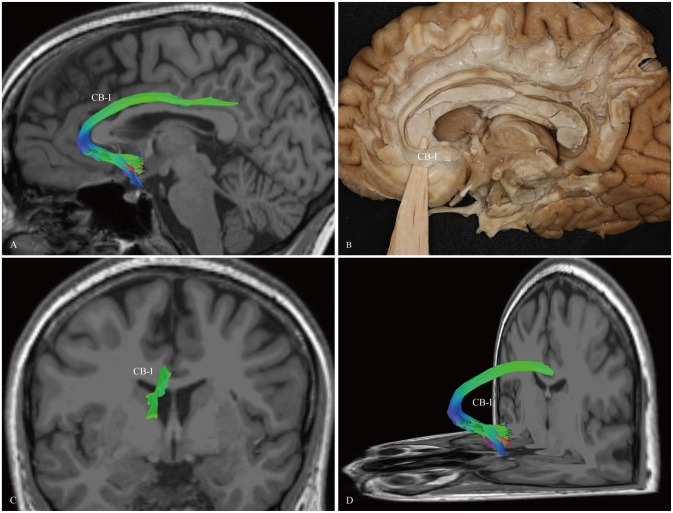
***In vivo* fiber tractography and fiber dissection of the CB-I. (A,B)** The CB-I runs from the subrostral areas in the orbital-frontal cortex anteriorly, then arches through almost 180° around the genu of the CC. **(C,D)** After that, the fibers continue above the body of the CC to the precuneus and splenium of the CC.

**Figure 4:**
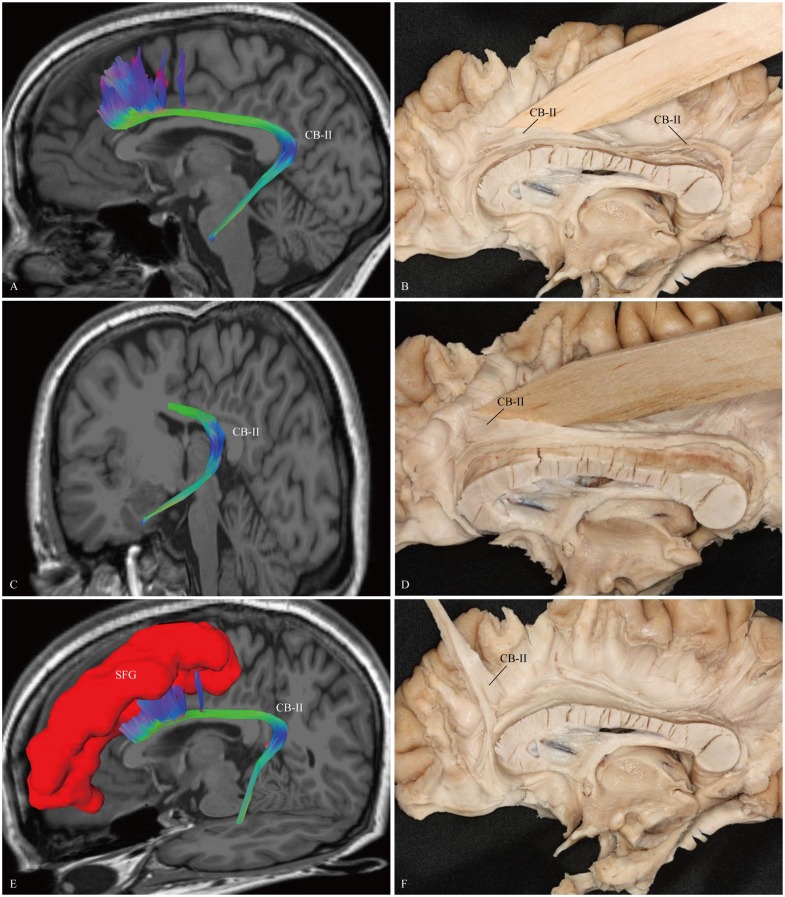
***In vivo* fiber tractography and fiber dissection of the CB-II. (A–C)** The CB-II originates from the para-hippocampal gyrus, arches around the splenium, then extends longitudinally above the CC. **(D–F)** The fibers continue anteriorly, lastly connect with the medial aspect of the SFG. SFG, superior frontal gyrus.

**Figure 5:**
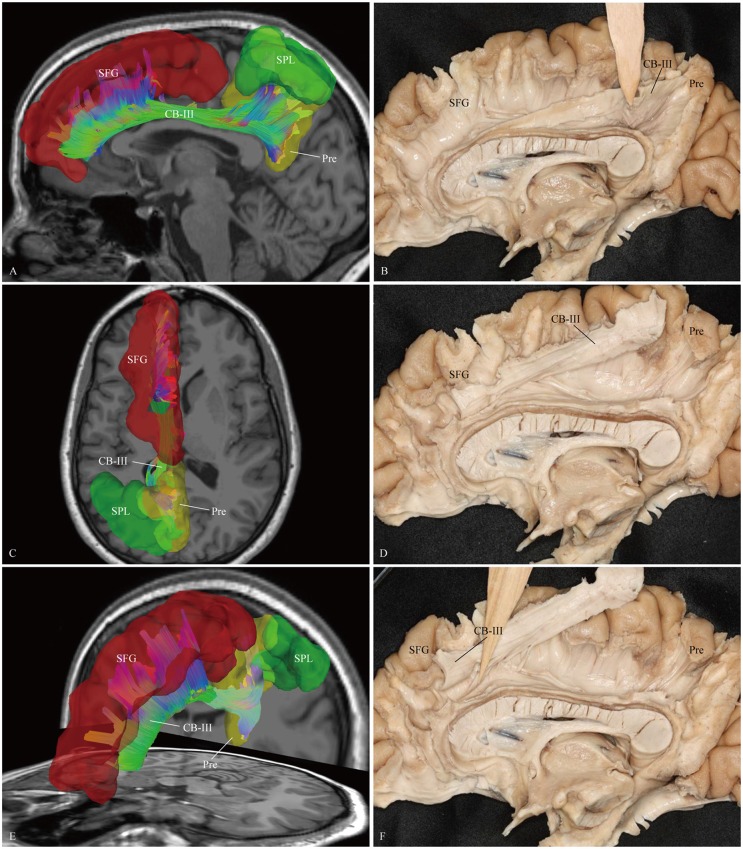
***In vivo* fiber tractography and fiber dissection of the CB-III. (A–D)** The CB-III originates from the superior parietal lobule (SPL) and precuneus, then extends along the trunk of the CC. **(E,F)** Finally, the fibers associate with the anterior and middle parts of the medial aspect of the SFG. SFG, superior frontal gyrus; SPL, superior parietal lobule; Pre, precuneus.

**Figure 6:**
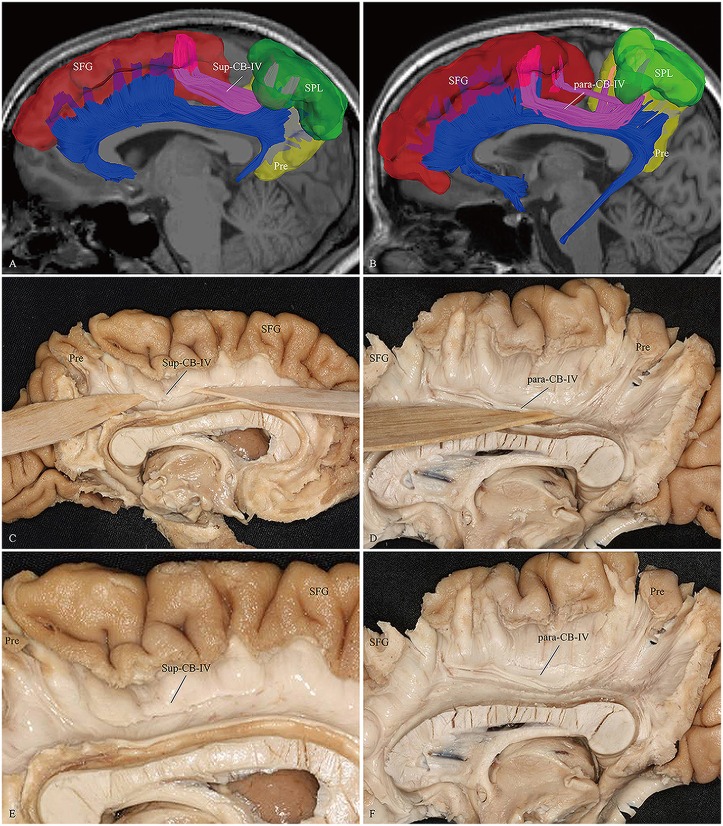
***In vivo* fiber tractography and fiber microdissection of the CB-IV.** The CB-IV is a short and minor tract that connects the SPL and precuneus with the supplementary and premotor areas. **(A,C,E)** The CB-IV (purple color) can be defined as supra-cingulate location with the other segments (blue color). **(B,D,F)** The CB-IV (purple color) can be defined as a para-cingulate location with the other segments (blue color). SFG, superior frontal gyrus; SPL, superior parietal lobule; Pre, precuneus.

**Figure 7:**
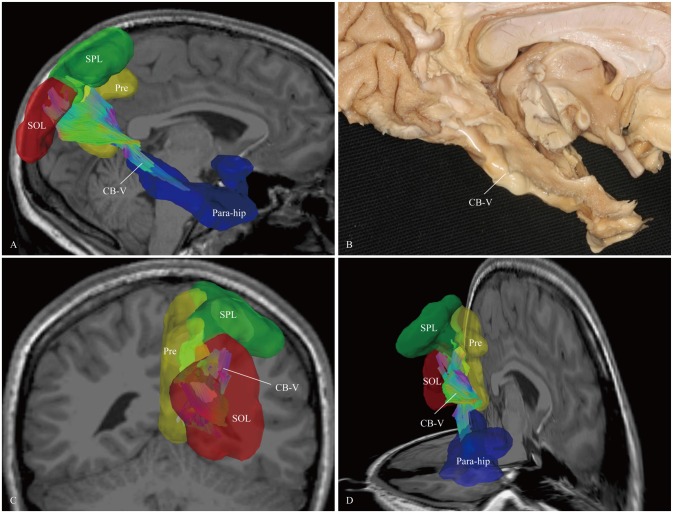
***In vivo* fiber tractography and fiber microdissection of the CB-V. (A,B)** The CB-V extends along the para-hippocampal gyrus, **(C,D)** then fans out to the parietal and occipital lobes. The scattered fibers in the parietal and occipital lobes are failed to be identified in fiber dissection. Para-hip, para-hippocampal gyrus; SPL, superior parietal lobule; Pre, precuneus; SOL, superior occipital lobule.

**Figure 8:**
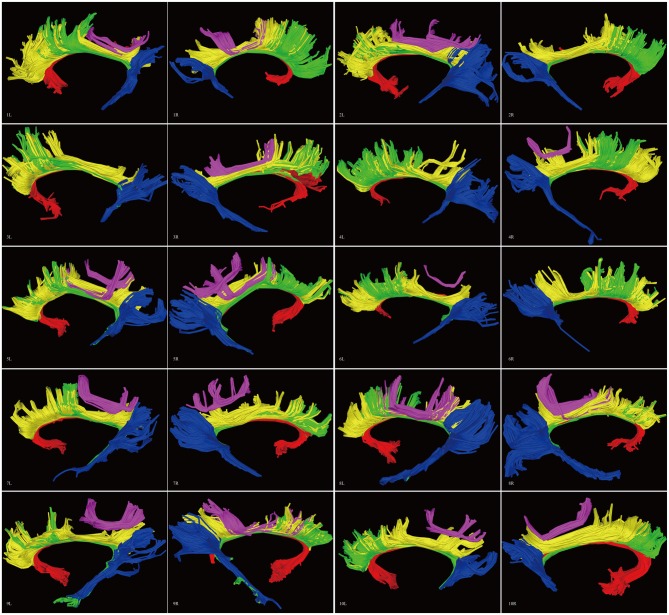
**Diffusion spectrum imaging (DSI) tractography of the CB in 20 hemispheres of 10 subjects on sagittal view.** Order: subject 1, 2, 3, 4, 5, 6, 7, 8, 9, 10. Left and right CB had a similar location, shape and trajectory in all 20 hemispheres. The CB-IV can be delineated in 16 out of 20 hemispheres. On further analysis of all subjects, the CB-IV is parallel and adjacent to the others in 7 out of 16 hemispheres. In the remaining nine hemispheres, the CB-IV is located above the others in a sup-CB pattern. Red color, CB-I; green color, CB-II; yellow color, CB-III; purple color, CB-IV; blue color, CB-V; L, left; R, right.

The CB-I ran from the orbital-frontal cortex, arched around the genu of the CC, then connected with the precuneus and splenium (Figure 3). Both the CB-II and the CB-V stemmed from the para-hippocampal gyrus. The CB-II associated with the medial aspect of the superior frontal gyrus (SFG; Figure 4), while the CB-V fanned out to the parietal and occipital lobes (Figure 7). The CB-III was the largest of the fiber bundles in the CB system and was identified in all hemispheres. These fibers originated from the precuneus and terminated at the medial aspect of the SFG (Figure 5). The CB-IV ran in two different patterns, the para-cingulate (para-CB) and the supra-cingulate (sup-CB; Figure 6). Further detailed analysis showed that left and right CB had a similar location, shape and trajectory in all 20 hemispheres (Figure 8).

#### Template Tractography Findings

**Figure 9:**
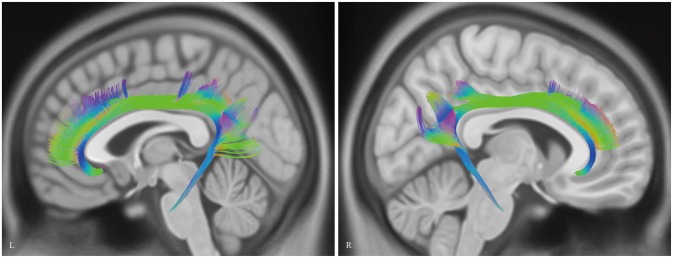
***In vivo* fiber tractography of the CB on Human Connectome Project (HCP-488) template.** CB, cingulum bundle; L, left; R, right.

Analysis of the HCP-488 template showed similar results to the sample subject findings above (Figure 9).

#### Fiber Dissection Findings

We first exposed the fibers originating from the precuneus that accounted for the largest part in the CB. These fibers originated from the precuneus, ran just above the CC and terminated at the medial aspect of the SFG (Figure 5). Then these fibers were removed, and the dissection was continued anteriorly. We identified two groups of fibers in the front area. One was connected to the medial aspect of the SFG, while the other arched inferiorly almost 180° around the front of the genu of the CC and ended in the subcallosal gyrus and the paraterminal gyrus (Figures 3, 4). Both bodies of these two groups of fibers encircled the CC and extended posteriorly to the splenium. Thereafter, we identified the fibers that connected the precuneus with the supplementary and premotor areas. The two different patterns (para-CB and sup-CB) were both found in the two cerebral hemispheres (Figure 6).

The last step was to expose the para-hippocampal subdivision. After peeling off the gray matter of the para-hippocampal gyrus, we identified the fibers forward to the anterior para-hippocampal region. We made all efforts to expose the portion that fanned out to the occipital lobes. However, we could not identify these scattered fibers in this multiple fiber-crossing area, which may have been due to the quality of the specimens or the limitations of the fiber dissection technique (Figure 7).

**Figure 10:**
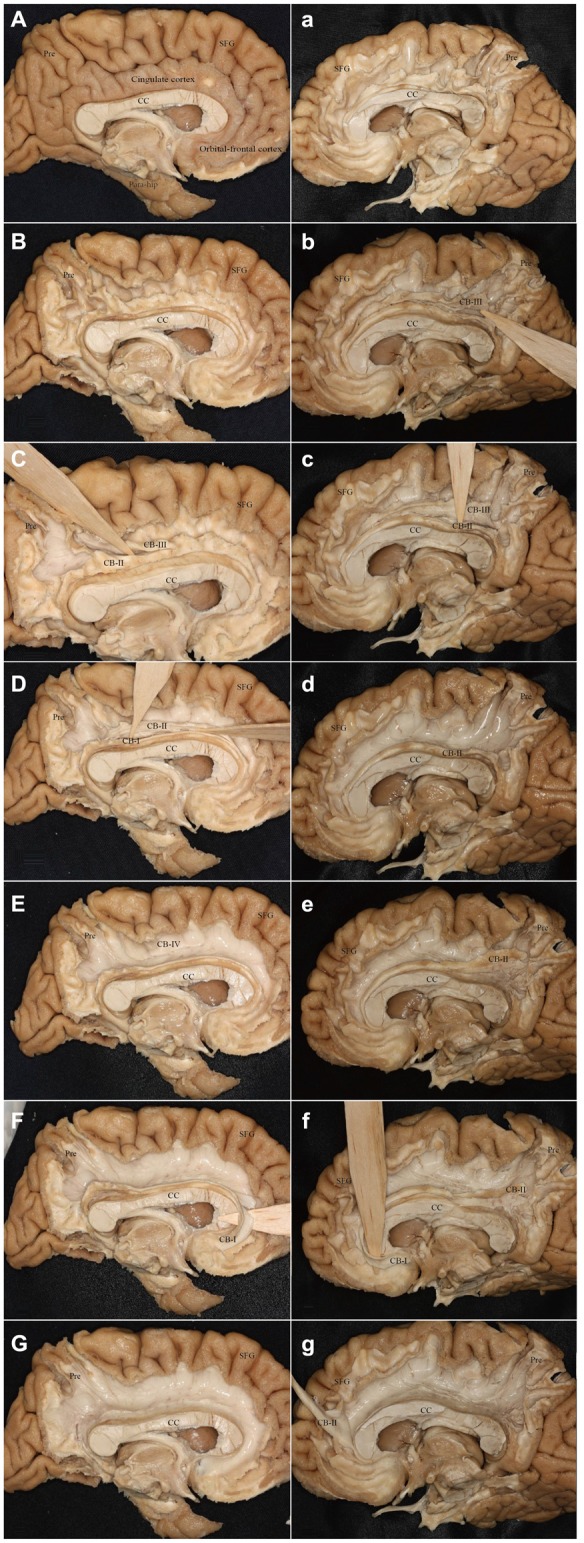
**Step-by-step fiber dissection of the CB (one left hemisphere and one right hemisphere). (A)** Medial surface of the left hemisphere after removing of vessels, arachnoid and pia-matter. **(B)** Removing the cortex within the depth of the sulci only, with preservation of the cortex of the hemispheres surface of the gyri. **(C)** Identifying the CB-III fibers that originate from the precuneus and extend above the CC. **(D)** Separating the fibers of the CB-I and CB-II above the CC. **(E)** After peeling off the CB-I, CB-II and CB-III, the supra-cingulate CB-IV can be visualized. **(F)** Identifying the CB-I fibers that originate from the subrostral areas. **(G)** Peeling off all the subcomponents. **(a)** Removing the cortex within the depth of the sulci only, with preservation of the cortex of the hemispheres surface of the gyri. **(b,c)** Separating the fibers of the CB-III and CB-II above the CC. **(d)** After peeling off the CB-III. **(e)** The body fibers of the CB-I and CB-II encircles above the CC (It is hard to separate them). **(f)** Identifying the CB-I fibers that originate from the subrostral areas. **(g)** The CB-II connects with the medial aspect of the SFG. Para-hip, para-hippocampal gyrus; Pre, precuneus; SFG, superior frontal gyrus; CC, corpus callosum.

It was hard to reveal all the subdivisions and identify the whole connections of the subcomponents in one hemisphere. For this, we showed two different hemispheres (one right and one left) for the step-by-step fiber dissection (Figure 10).

### Spatial Relationship of the Subcomponents of the CB

**Figure 11:**
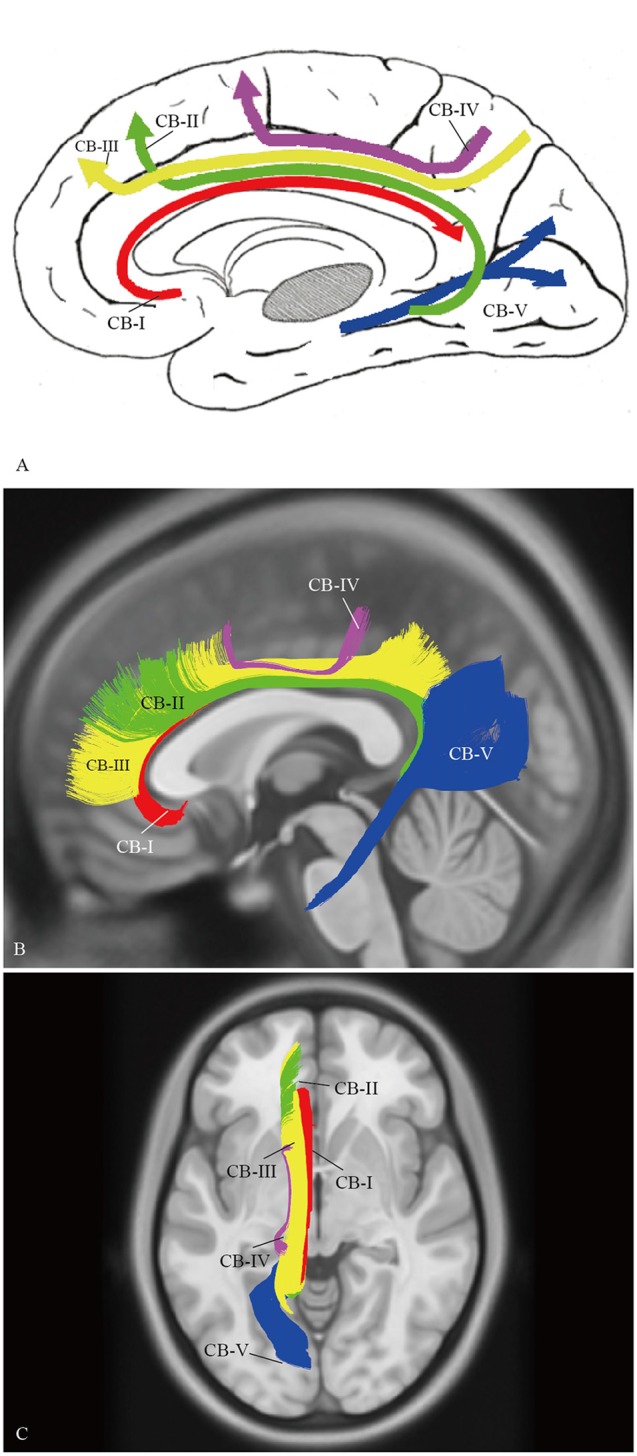
**Spatial Relationship of the subcomponents on HCP-488 template. (A)** A complete schematic map of the five segments. **(B)** Depicting five segments of CB on the HCP-488 template T_1_ sagittal view. The CB-I snugly curves around the genu of the CC and spreads into the orbital-frontal cortex as the lowest subdivision. For the projective scope in the medial aspect of the SFG, the CB-III is obviously larger than that of the CB-II. The body section of the CB-II extends along the bottom of the CB-III, while the caudal section is slightly more medial than the CB-V, despite their overlaps. **(C)** Axial view. The CB-I is closest to the midline in all the segments. The CB-IV is the smallest fiber tract in the CB system. It is situated superficially lateral to the CB-III.

A complete schematic map of the five segments was shown in Figure 11A. On representative T_1_ sections, the CB-I was the lowest and was the closest to the midline in the subcomponents (Figure 11B). The CB-II extended along the bottom of the CB-III, more medial than the CB-V (Figure 11B). The CB-IV was situated superficially lateral to the CB-III (Figure 11C). The CB-V was situated slightly more lateral (Figure 11B).

## Discussion

In the present study, we investigated the trajectory, associated architecture and anatomical connectivity of the CB in 10 subjects and a template of 488 subjects using HDFT. Compared to previous DTI reports, our study not only presented architectures of the CB with significantly higher resolution, but also demonstrated a more complete connectivity pattern of the CB. In addition, we also investigated whether there were discernible structural subdivisions in the CB based on white matter corticocortical connections. We found that there were five potentially different subcomponents within the CB, which were completely attained in both the small independent sample (*n* = 10) as well as the larger template sample (*n* = 488). The findings of fiber dissection were consistent with that of *in vivo* tractography.

The CB was originally described by Vicq d’Azyr in 1786 as one of the more complex white matter tracts in the human brain. As the CB runs through a large extent of the brain, it had been reported that any changes in specific subregions of the CB probably resulted in different functional disorders (Jang et al., 2013). Previously, it had been widely accepted that the CB was segmented into anterior-posterior-inferior portions. However, in some autoradiographic tracing studies of the rhesus monkeys, three distinct fiber components of the CB were found. They were segmented as the connections of different cortical and subcortical regions (Mufson and Pandya, 1984). In other animal studies, both the afferent or efferent fibers were reported to occupy specific parts of the CB, connecting various cortical and subcortical regions (Vogt and Pandya, 1987; Kobayashi and Amaral, 2007). Therefore, we hypothesized that the CB system in humans might show similar corticocortical segmentation patterns. Recently, a DTI study in 20 healthy humans suggested that the CB could be subdivided into three different components (Jones et al., 2013). However, these findings needed independent confirmation at both the neuroimaging and neuroanatomical whole-brain level.

In this study, we reconstructed the CB based on the cortical ROIs defined by the Automated Anatomical Labeling (Desikan et al., 2006). The anatomic subregions are automatically generated in light of individuality which are viewed as ideal for generating tracts. After reconstructing the whole bundle, we traced back the fibers according to the cortex landmarks. Furthermore, HDFT, a high-angular-resolution-based approach employing DSI reconstructed by GQI, has been proven to be able to allow tracing from one cortical region to other cortical or subcortical regions through complex crossings areas, providing detailed evidence of the cortical site of origin or termination of the fibers without the need for approximation (Fernandez-Miranda, 2013; Fernández-Miranda et al., 2015; Wu et al., 2016). In addition, our diffusion imaging technique involves a 43 min, 176 slices and 257-direction scan, as compared with the 26 min, 60 slices and 60-direction scan used by Jones et al. (2013). All of the above are the main advantages that keep our results accurate and convincing.

On the other hand, rather than tractography results, fiber dissections can provide a more intuitive observation to represent valuable information on the anatomy of the fiber tracts. To our knowledge, it is for the first time that the complex composition of the CB is assessed in detail by fiber dissection. The two methods (tractography and fiber dissection) cross-validated here, increase the confidence that our result is not an artifact and provides a stronger support for the described connectivity of the tracing fiber than when either of the method was used alone.

Our tractographic and anatomic results suggest that the CB is not a single pathway, but rather a composition of five distinct subcomponents of white matter at different levels. We assume that each subcomponent not only contributes to the formation of a CB fiber system, but also retains its own characteristics. According to its interconnected cortical regions, we provided a detailed analysis of each subcomponent in the CB. We also described the potential role of these distinct subdivisions based on cross-species studies of monkey fiber connections, fiber trajectories from our observations and lesion-deficit correlative analysis in the human brain.

### CB-I

The subgenual cingulate region (Brodmann area 25) and the orbital frontal (area 11) were suggested to play a critical role in modulating negative mood states (Seminowicz et al., 2004). In fact, previous monkey tract tracing studies showed that the connections from the orbital cortex formed at a distinct network within cingulate limbic structures (Carmichael and Price, 1995, 1996). Combining the polarized light imaging (PLI) and the macroscopical fiber dissection in the human brain, Axer et al. (2011) reported that, after running above the CC, the pregenual part of the CB curved around the genu, and then spread into the orbitofrontal white matter. All of the above are consistent with our descriptions of the CB-I (Figure 3).

The functional role of the CB-I remains to be ascertained. Several studies revealed that activation of the perigenual cortex and PCC might participate in affective responses to noxious stimuli (Ballantine et al., 1967; Casey et al., 1994; Vogt et al., 1996). Using electrical stimulation of the subgenual cingulate white matter on six patients, Mayberg et al. (2005) suggested that disrupting focal pathological activity in limbic-cortical circuits could effectively reverse symptoms in otherwise treatment-resistant depression. The functional imaging study found that the fiber microstructural integrity connecting the genu and splenium showed the highest significant relationship with global cognitive function and verbal memory performance (Tuladhar et al., 2015).

### CB-II

Previous anatomical studies of monkeys have confirmed a long fiber component connecting with medial temporal and cingulate limbic structures, named as the “medial” network (Kondo et al., 2003). This system originates from the retrosplenial, para-hippocampal, and perirhinal cortices, extends above the CC, then ends in the medial frontal cortex (Saleem et al., 2008). The monkey “medial” network is similar to our CB-II observations in the human brain (Figure 4).

Path integration uses constant updation of the navigator’s representation of position and orientation during movement without using landmarks, and often involves tracking a start or home location (Byrne et al., 2007). Using a novel loop closure task in 31 participants, Chrastil et al. (2015) found that the hippocampus, RSC, para-hippocampal cortex and medial prefrontal cortex (mPFC) were recruited during successful path integration. Interestingly, in our results, the course of fiber tractography of the CB-II associates with all these brain regions, suggests a potential role of the CB-II in supporting path integration.

### CB-III

Our observations suggest that the CB-III is situated within the white matter of the superior parietal lobule (SPL) (area 7), the precuneus (medial area 7), the medial part of SFG (areas 8, 9 and 32) and the supplementary motor area (area 6; Figure 5). There is a close spatial relationship between the CB-III and the CB-II, however, the CB-III is clearly discernible from the CB as a distinctive subcomponent in our tractography analysis (Figure 11). The observations shown in post-mortem also correspond to the DSI results. But, it is not feasible for us to extrapolate a similar connection with the monkey experimental material. The evolution of neurocranial morphology in *Homo sapiens* is characterized by bulging of the parietal region, a feature unique to our species. Comparisons between human brains and macaque brains have shown that there are species-specific differences in the parietal cortex, such as the structural and functional connections of the intraparietal sulcal cortex (Orban et al., 2006; Scheperjans et al., 2008). Moreover, similar comparative neuroanatomical and paleontological evidence has suggested that precuneus expansion is also a neurological specialization of *Homo sapiens* (Bruner et al., 2016).

The CB-III is the largest-volume tract in the CB system. Given its multiple interconnected cortical regions and evolutional variation in brain shape among humans, we speculate that the CB-III may be the most significant part of the multi-functional CB tract, which is associated with the recent human cognitive specializations. This proposition can be supported by some functional studies. For example, white matter connectivity between the pre-SMA and the CB was associated with response conflict in healthy human subjects (Yamamoto et al., 2015). Huang et al. (2013, 2014) also found that the microstructural integrity of the white matter connecting the anterior cingulate area to the precuneus and medial superior frontal areas reflected the level of neurophysiologic dependence in tobacco withdrawal syndrome. Furthermore, several studies have proved that performance in attention-demanding cognitive tasks was related to the neural structure connecting the PCC, medial and lateral parietal cortex, and the mPFC (Raichle et al., 2001; McKiernan et al., 2003; Fox et al., 2005).

### CB-IV

The CB-IV is the shortest subdivision in the CB system that connects the superior parietal lobe (area 7) and precuneus (medial area 7) with the supplementary and premotor areas (areas 8, 9 and 32). We identified both para-cingulate (para-CB) and supra-cingulate (supra-CB) patterns in fiber dissection and DSI tractography (Figure 6). Interestingly, the CB-IV connection is similar to the previously described superior longitudinal fasciculus I (SLF-I). Kamali et al. (2014a) assessed the trajectory and connections of the SLF-I in five subjects based on DTI technique. They described that the SLF-I located closely with the CB, which could be distinguished from the adjacent CB clearly. Another study by Jang and Hong (2012) yielded similar results, though they only reconstructed the SLF-I using the cortical ROIs without evaluating the role of CB. As previous studies have revealed that some fibers of the CB originated from the SPL and precuneus to the frontal region, our results showed that it was difficult to separate the SLF-I from the CB, especially in the para-CB location. Thus, we speculated that few fibers of the CB had been somewhat assigned to the SLF-I artificially in previous DTI studies (Thiebaut de Schotten et al., 2011; Kamali et al., 2014a,b).

Recently, as its anatomical location was much closer to the cingulum fiber system (adjacent or just above) than to the superior longitudinal fascicle, Wang et al. (2016) suggested that this fiber bundle should be considered as part of the CB system rather than the SLF system. However, they failed to verify this assumption in fiber dissection. Our results provide important visible evidence to support this hypothesis for the first time.

### CB-V

In the past decades, neuroscientists have gained insight into the anatomy of the para-hippocampal cingulum (CB-V) in humans based on tractographic and fiber anatomical studies (Fernández-Miranda et al., 2008; Jones et al., 2013). Similar connections have been found in non-human primates (Pandya et al., 1981; Kobayashi and Amaral, 2007). In agreement with previous studies of the para-hippocampal cingulum, we found a tract running from the medial temporal lobe to the parietal and occipital lobes (Figure 7; Sethi et al., 2015). Compared to previous reports, our results showed several important anatomical characteristics of the CB-V. First, the CB-V is situated slightly more lateral than the other subdivisions. Second, although the CB-V runs side-by-side with the CB-III, it extends further forward within the medial temporal lobe than the CB-III (Figure 11).

The para-hippocampal cingulum white matter connections are a target of DTI studies for early diagnosis in AD (Wisse et al., 2015). It is possible that the brain regions associated with the CB-V are the earliest to exhibit neuronal degeneration. Killiany et al. (2002) reported that the entorhinal cortex displayed early degeneration in AD patients, while the extent of hippocampal atrophy influenced episodic memory performance in AD (Ezzati et al., 2015). A relationship between para-hippocampal cingulum integrity and the decline in various domains of cognitive function, such as spatial navigation and attention-shifting tasks, was also observed in AD patients (Lin et al., 2014). For this, we assume that the CB-V constitutes a “control” pathway supporting memory, executive function and other cognitive functions (Buckner, 2004; Seeley et al., 2007).

There are several limitations in our study. First although fiber dissection can provide key macroscopic information on fiber tract anatomy, it has limited value for the study of white matter connectivity in areas of crossing fibers. Second, the subject-specific approach is performed in only 10 subjects, whereas the HCP-488 template represents an averaged map. More subjects are required to provide a more detailed understanding of CB pathway variability in the human brain, including the effects of gender and age. Nevertheless, our findings open up a new perspective for subdividing the CB, higher resolution tractography or other methods maybe uncover further dissociations in future.

## Conclusion

Using fiber dissection and HDFT tractography, our study identified the whole CB and its five subcomponents in the human brain for the first time. Our findings provide clear evidence that the CB is not a single entity, but rather a composition of distinct subdivisions at different levels. This segmentation of the CB will offer new insights into the associated structural-functional and anatomical-clinical changes in the CB system.

## Author Contributions

Conceived and designed the experiments: YuW and YiW. Performed the experiments: YuW, YiW and YoW. Analyzed the data and image processing: YuW and DS. Collected the biopsy samples: YiW and YoW. Contributed reagents/materials/analyses tools: SO and YiW. Wrote the article and revised the manuscript: YuW, SO and YiW.

## Conflict of Interest Statement

The authors declare that the research was conducted in the absence of any commercial or financial relationships that could be construed as a potential conflict of interest.

## Footnotes

^1^http://dsi-studio.labsolver.org

## Abbreviations

CB, cingulum bundle; DSI, diffusion spectrum imaging; DTI, diffusion tensor imaging; GQI, Generalized q-sampling imaging; HDFT, high definition fiber tracking; QA, quantitative anisotropy; ROA, Region of avoidance; ROI, region of interest.

## References

[B1] Adnan A. Barnett A. Moayedi M. McCormick C. Cohn M. McAndrews M. P. (2016). Distinct hippocampal functional networks revealed by tractography-based parcellation. Brain Struct. Funct. 221, 2999–3012. 10.1007/s00429-015-1084-x 26206251

[B2] Agrawal A. Kapfhammer J. P. Kress A. Wichers H. Deep A. Feindel W.. (2011). Josef Klingler’s models of white matter tracts: influences on neuroanatomy, neurosurgery and neuroimaging. Neurosurgery 69, 238–252; discussion 252–234. 10.1227/NEU.0b013e318214ab79 21368687

[B3] Axer H. Beck S. Axer M. Schuchardt F. Heepe J. Flucken A.. (2011). Microstructural analysis of human white matter architecture using polarized light imaging: views from neuroanatomy. Front. Neuroinform. 5:28. 10.3389/fninf.2011.00028 22110430 PMC3215979

[B4] Ballantine H. T. Jr. Cassidy W. L. Flanagan N. B. Marino R. Jr. (1967). Stereotaxic anterior cingulotomy for neuropsychiatric illness and intractable pain. J. Neurosurg. 26, 488–495. 10.3171/jns.1967.26.5.0488 5337782

[B5] Benes F. M. (1998). Model generation and testing to probe neural circuitry in the cingulate cortex of postmortem schizophrenic brain. Schizophr. Bull. 24, 219–230. 10.1093/oxfordjournals.schbul.a033322 9613622

[B6] Bruner E. Preuss T. M. Chen X. Rilling J. K. (2016). Evidence for expansion of the precuneus in human evolution. Brain Struct. Funct. [Epub ahead of print]. 10.1007/s00429-015-1172-y 26725108 PMC4930733

[B7] Buckner R. L. (2004). Memory and executive function in aging and AD: multiple factors that cause decline and reserve factors that compensate. Neuron 44, 195–208. 10.1016/j.neuron.2004.09.006 15450170

[B8] Byrne P. Becker S. Burgess N. (2007). Remembering the past and imagining the future: a neural model of spatial memory and imagery. Psychol. Rev. 114, 340–375. 10.1037/0033-295x.114.2.340 17500630 PMC2678675

[B9] Carmichael S. T. Price J. L. (1995). Limbic connections of the orbital and medial prefrontal cortex in macaque monkeys. J. Comp. Neurol. 363, 615–641. 10.1002/cne.903630408 8847421

[B10] Carmichael S. T. Price J. L. (1996). Connectional networks within the orbital and medial prefrontal cortex of macaque monkeys. J. Comp. Neurol. 371, 179–207. 10.1002/(SICI)1096-9861(19960722)371:2<179::AID-CNE1>3.0.CO;2-# 8835726

[B11] Casey K. L. Minoshima S. Berger K. L. Koeppe R. A. Morrow T. J. Frey K. A. (1994). Positron emission tomographic analysis of cerebral structures activated specifically by repetitive noxious heat stimuli. J. Neurophysiol. 71, 802–807. 8176441 10.1152/jn.1994.71.2.802

[B12] Catani M. Thiebaut de Schotten M. (2008). A diffusion tensor imaging tractography atlas for virtual *in vivo* dissections. Cortex 44, 1105–1132. 10.1016/j.cortex.2008.05.004 18619589

[B13] Catheline G. Periot O. Amirault M. Braun M. Dartigues J. F. Auriacombe S.. (2010). Distinctive alterations of the cingulum bundle during aging and Alzheimer’s disease. Neurobiol. Aging 31, 1582–1592. 10.1016/j.neurobiolaging.2008.08.012 18829135

[B14] Choo I. H. Lee D. Y. Oh J. S. Lee J. S. Lee D. S. Song I. C.. (2010). Posterior cingulate cortex atrophy and regional cingulum disruption in mild cognitive impairment and Alzheimer’s disease. Neurobiol. Aging 31, 772–779. 10.1016/j.neurobiolaging.2008.06.015 18687503

[B15] Chrastil E. R. Sherrill K. R. Hasselmo M. E. Stern C. E. (2015). There and back again: hippocampus and retrosplenial cortex track homing distance during human path integration. J. Neurosci. 35, 15442–15452. 10.1523/JNEUROSCI.1209-15.2015 26586830 PMC6605486

[B16] Concha L. Gross D. W. Beaulieu C. (2005). Diffusion tensor tractography of the limbic system. AJNR Am. J. Neuroradiol. 26, 2267–2274. 16219832 PMC7976161

[B17] Desikan R. S. Segonne F. Fischl B. Quinn B. T. Dickerson B. C. Blacker D.. (2006). An automated labeling system for subdividing the human cerebral cortex on MRI scans into gyral based regions of interest. Neuroimage 31, 968–980. 10.1016/j.neuroimage.2006.01.021 16530430

[B18] Ezzati A. Katz M. J. Lipton M. L. Zimmerman M. E. Lipton R. B. (2015). Hippocampal volume and cingulum bundle fractional anisotropy are independently associated with verbal memory in older adults. Brain Imaging Behav. [Epub ahead of print]. 10.1007/s11682-015-9452-y 26424564 PMC4816657

[B19] Fernandez-Miranda J. C. (2013). Editorial: beyond diffusion tensor imaging. J. Neurosurg. 118, 1363–1365; discussion 1365–1366. 10.3171/2012.10.JNS121800 23540267

[B20] Fernandez-Miranda J. C. Pathak S. Engh J. Jarbo K. Verstynen T. Yeh F. C.. (2012). High-definition fiber tractography of the human brain: neuroanatomical validation and neurosurgical applications. Neurosurgery 71, 430–453. 10.1227/NEU.0b013e3182592faa 22513841

[B21] Fernández-Miranda J. C. Rhoton A. L. Jr. Alvarez-Linera J. Kakizawa Y. Choi C. de Oliveira E. P. (2008). Three-dimensional microsurgical and tractographic anatomy of the white matter of the human brain. Neurosurgery 62, 989–1026; discussion 1026–1028. 10.1227/01.neu.0000333767.05328.49 18695585

[B22] Fernández-Miranda J. C. Wang Y. Pathak S. Stefaneau L. Verstynen T. Yeh F. C. (2015). Asymmetry, connectivity and segmentation of the arcuate fascicle in the human brain. Brain Struct. Funct. 220, 1665–1680. 10.1007/s00429-014-0751-7 24633827

[B23] Fox M. D. Snyder A. Z. Vincent J. L. Corbetta M. Van Essen D. C. Raichle M. E. (2005). The human brain is intrinsically organized into dynamic, anticorrelated functional networks. Proc. Natl. Acad. Sci. U S A 102, 9673–9678. 10.1073/pnas.0504136102 15976020 PMC1157105

[B24] Haznedar M. M. Buchsbaum M. S. Hazlett E. A. Shihabuddin L. New A. Siever L. J. (2004). Cingulate gyrus volume and metabolism in the schizophrenia spectrum. Schizophr. Res. 71, 249–262. 10.1016/j.schres.2004.02.025 15474896

[B25] Heilbronner S. R. Haber S. N. (2014). Frontal cortical and subcortical projections provide a basis for segmenting the cingulum bundle: implications for neuroimaging and psychiatric disorders. J. Neurosci. 34, 10041–10054. 10.1523/JNEUROSCI.5459-13.2014 25057206 PMC4107396

[B26] Huang W. DiFranza J. R. Kennedy D. N. Zhang N. Ziedonis D. Ursprung S.. (2013). Progressive levels of physical dependence to tobacco coincide with changes in the anterior cingulum bundle microstructure. PLoS One 8:e67837. 10.1371/journal.pone.0067837 23861816 PMC3701580

[B27] Huang W. King J. A. Ursprung W. W. Zheng S. Zhang N. Kennedy D. N.. (2014). The development and expression of physical nicotine dependence corresponds to structural and functional alterations in the anterior cingulate-precuneus pathway. Brain Behav. 4, 408–417. 10.1002/brb3.227 24944870 PMC4055191

[B28] Jang S. H. Hong J. H. (2012). The anatomical characteristics of superior longitudinal fasciculus I in human brain: diffusion tensor tractography study. Neurosci. Lett. 506, 146–148. 10.1016/j.neulet.2011.10.069 22085696

[B29] Jang S. H. Kim S. H. Kim O. R. Byun W. M. Kim M. S. Seo J. P.. (2013). Cingulum injury in patients with diffuse axonal injury: a diffusion tensor imaging study. Neurosci. Lett. 543, 47–51. 10.1016/j.neulet.2013.02.058 23562507

[B30] Jansons K. M. Alexander D. C. (2003). Persistent angular structure: new insights from diffusion MRI data. Dummy version. Inf. Process. Med. Imaging 18, 672–683. 10.1007/978-3-540-45087-0_56 15344497

[B31] Jones D. K. Christiansen K. F. Chapman R. J. Aggleton J. P. (2013). Distinct subdivisions of the cingulum bundle revealed by diffusion MRI fibre tracking: implications for neuropsychological investigations. Neuropsychologia 51, 67–78. 10.1016/j.neuropsychologia.2012.11.018 23178227 PMC3611599

[B32] Kamali A. Flanders A. E. Brody J. Hunter J. V. Hasan K. M. (2014a). Tracing superior longitudinal fasciculus connectivity in the human brain using high resolution diffusion tensor tractography. Brain Struct. Funct. 219, 269–281. 10.1007/s00429-012-0498-y 23288254 PMC3633629

[B33] Kamali A. Sair H. I. Radmanesh A. Hasan K. M. (2014b). Decoding the superior parietal lobule connections of the superior longitudinal fasciculus/arcuate fasciculus in the human brain. Neuroscience 277, 577–583. 10.1016/j.neuroscience.2014.07.035 25086308

[B34] Killiany R. J. Hyman B. T. Gomez-Isla T. Moss M. B. Kikinis R. Jolesz F.. (2002). MRI measures of entorhinal cortex vs. hippocampus in preclinical AD. Neurology 58, 1188–1196. 10.1212/wnl.58.8.1188 11971085

[B35] Kobayashi Y. Amaral D. G. (2007). Macaque monkey retrosplenial cortex: III. Cortical efferents. J. Comp. Neurol. 502, 810–833. 10.1002/cne.21346 17436282

[B36] Kondo H. Saleem K. S. Price J. L. (2003). Differential connections of the temporal pole with the orbital and medial prefrontal networks in macaque monkeys. J. Comp. Neurol. 465, 499–523. 10.1002/cne.10842 12975812

[B37] Lawes I. N. Barrick T. R. Murugam V. Spierings N. Evans D. R. Song M.. (2008). Atlas-based segmentation of white matter tracts of the human brain using diffusion tensor tractography and comparison with classical dissection. Neuroimage 39, 62–79. 10.1016/j.neuroimage.2007.06.041 17919935

[B38] Lin Y. C. Shih Y. C. Tseng W. Y. Chu Y. H. Wu M. T. Chen T. F.. (2014). Cingulum correlates of cognitive functions in patients with mild cognitive impairment and early Alzheimer’s disease: a diffusion spectrum imaging study. Brain Topogr. 27, 393–402. 10.1007/s10548-013-0346-2 24414091

[B39] Ludwig E. Klingler J. (1965). Atlas Cerebri Humani. The Inner Structure of the Brain Demonstrated on the Basis of Macroscopical Preparations. Boston, MA: Little Brown.

[B40] Martino J. De Witt Hamer P. C. Vergani F. Brogna C. de Lucas E. M. Vazquez-Barquero A.. (2011). Cortex-sparing fiber dissection: an improved method for the study of white matter anatomy in the human brain. J. Anat. 219, 531–541. 10.1111/j.1469-7580.2011.01414.x 21767263 PMC3196758

[B41] Mayberg H. S. Lozano A. M. Voon V. McNeely H. E. Seminowicz D. Hamani C.. (2005). Deep brain stimulation for treatment-resistant depression. Neuron 45, 651–660. 10.1016/j.neuron.2005.02.014 15748841

[B42] McKiernan K. A. Kaufman J. N. Kucera-Thompson J. Binder J. R. (2003). A parametric manipulation of factors affecting task-induced deactivation in functional neuroimaging. J. Cogn. Neurosci. 15, 394–408. 10.1162/089892903321593117 12729491

[B43] Mufson E. J. Pandya D. N. (1984). Some observations on the course and composition of the cingulum bundle in the rhesus monkey. J. Comp. Neurol. 225, 31–43. 10.1002/cne.902250105 6725639

[B44] Orban G. A. Claeys K. Nelissen K. Smans R. Sunaert S. Todd J. T.. (2006). Mapping the parietal cortex of human and non-human primates. Neuropsychologia 44, 2647–2667. 10.1016/j.neuropsychologia.2005.11.001 16343560

[B45] Pandya D. N. Van Hoesen G. W. Mesulam M. M. (1981). Efferent connections of the cingulate gyrus in the rhesus monkey. Exp. Brain Res. 42, 319–330. 10.1007/bf00237497 6165607

[B46] Raichle M. E. MacLeod A. M. Snyder A. Z. Powers W. J. Gusnard D. A. Shulman G. L. (2001). A default mode of brain function. Proc. Natl. Acad. Sci. U S A 98, 676–682. 10.1073/pnas.98.2.676 11209064 PMC14647

[B47] Saleem K. S. Kondo H. Price J. L. (2008). Complementary circuits connecting the orbital and medial prefrontal networks with the temporal, insular and opercular cortex in the macaque monkey. J. Comp. Neurol. 506, 659–693. 10.1002/cne.21577 18067141

[B48] Scheperjans F. Hermann K. Eickhoff S. B. Amunts K. Schleicher A. Zilles K. (2008). Observer-independent cytoarchitectonic mapping of the human superior parietal cortex. Cereb. Cortex 18, 846–867. 10.1093/cercor/bhm116 17644831

[B49] Schmahmann J. D. Pandya D. N. (2006). Fiber Pathways of the Brain. New York, NY: Oxford UP.

[B50] Seeley W. W. Menon V. Schatzberg A. F. Keller J. Glover G. H. Kenna H.. (2007). Dissociable intrinsic connectivity networks for salience processing and executive control. J. Neurosci. 27, 2349–2356. 10.1523/JNEUROSCI.5587-06.2007 17329432 PMC2680293

[B51] Seminowicz D. A. Mayberg H. S. McIntosh A. R. Goldapple K. Kennedy S. Segal Z.. (2004). Limbic-frontal circuitry in major depression: a path modeling metanalysis. Neuroimage 22, 409–418. 10.1016/j.neuroimage.2004.01.015 15110034

[B52] Sethi A. Gregory S. Dell’Acqua F. Periche Thomas E. Simmons A. Murphy D. G.. (2015). Emotional detachment in psychopathy: involvement of dorsal default-mode connections. Cortex 62, 11–19. 10.1016/j.cortex.2014.07.018 25218645

[B53] Sexton C. E. Kalu U. G. Filippini N. Mackay C. E. Ebmeier K. P. (2011). A meta-analysis of diffusion tensor imaging in mild cognitive impairment and Alzheimer’s disease. Neurobiol. Aging 32, 2322.e5–2322.e18. 10.1016/j.neurobiolaging.2010.05.019 20619504

[B54] Sotiropoulos S. N. Jbabdi S. Xu J. Andersson J. L. Moeller S. Auerbach E. J.. (2013). Advances in diffusion MRI acquisition and processing in the Human Connectome Project. Neuroimage 80, 125–143. 10.1016/j.neuroimage.2013.05.057 23702418 PMC3720790

[B55] Thiebaut de Schotten M. Dell’Acqua F. Forkel S. J. Simmons A. Vergani F. Murphy D. G.. (2011). A lateralized brain network for visuospatial attention. Nat. Neurosci. 14, 1245–1246. 10.1038/nn.2905 21926985

[B56] Thiebaut de Schotten M. Dell’Acqua F. Valabregue R. Catani M. (2012). Monkey to human comparative anatomy of the frontal lobe association tracts. Cortex 48, 82–96. 10.1016/j.cortex.2011.10.001 22088488

[B57] Tuladhar A. M. van Norden A. G. de Laat K. F. Zwiers M. P. van Dijk E. J. Norris D. G.. (2015). White matter integrity in small vessel disease is related to cognition. Neuroimage Clin. 7, 518–524. 10.1016/j.nicl.2015.02.003 25737960 PMC4338206

[B58] Van Essen D. C. Smith S. M. Barch D. M. Behrens T. E. Yacoub E. Ugurbil K.. (2013). The WU-minn human connectome project: an overview. Neuroimage 80, 62–79. 10.1016/j.neuroimage.2013.05.041 23684880 PMC3724347

[B59] Vogt B. A. (2016). Midcingulate cortex: structure, connections, homologies, functions and diseases. J. Chem. Neuroanat. 74, 28–46. 10.1016/j.jchemneu.2016.01.010 26993424

[B62] Vogt B. A. Derbyshire S. Jones A. K. (1996). Pain processing in four regions of human cingulate cortex localized with co-registered PET and MR imaging. Eur. J. Neurosci. 8, 1461–1473. 10.1111/j.1460-9568.1996.tb01608.x 8758953

[B63] Vogt B. A. Finch D. M. Olson C. R. (1992). Functional heterogeneity in cingulate cortex: the anterior executive and posterior evaluative regions. Cereb. Cortex 2, 435–443. 10.1093/cercor/2.6.435-a 1477524

[B60] Vogt B. A. Laureys S. (2005). Posterior cingulate, precuneal and retrosplenial cortices: cytology and components of the neural network correlates of consciousness. Prog. Brain Res. 150, 205–217. 10.1016/s0079-6123(05)50015-3 16186025 PMC2679949

[B64] Vogt B. A. Nimchinsky E. A. Vogt L. J. Hof P. R. (1995). Human cingulate cortex: surface features, flat maps and cytoarchitecture. J. Comp. Neurol. 359, 490–506. 10.1002/cne.903590310 7499543

[B61] Vogt B. A. Pandya D. N. (1987). Cingulate cortex of the rhesus monkey: II. Cortical afferents. J. Comp. Neurol. 262, 271–289. 10.1002/cne.902620208 3624555

[B66] Wang Y. Fernández-Miranda J. C. Verstynen T. Pathak S. Schneider W. Yeh F. C. (2013). Rethinking the role of the middle longitudinal fascicle in language and auditory pathways. Cereb. Cortex 23, 2347–2356. 10.1093/cercor/bhs225 22875865

[B65] Wang X. Pathak S. Stefaneanu L. Yeh F. C. Li S. Fernandez-Miranda J. C. (2016). Subcomponents and connectivity of the superior longitudinal fasciculus in the human brain. Brain Struct. Funct. 221, 2075–2092. 10.1007/s00429-015-1028-5 25782434

[B67] Wedeen V. J. Hagmann P. Tseng W. Y. Reese T. G. Weisskoff R. M. (2005). Mapping complex tissue architecture with diffusion spectrum magnetic resonance imaging. Magn. Reson. Med. 54, 1377–1386. 10.1002/mrm.20642 16247738

[B68] Wedeen V. J. Wang R. P. Schmahmann J. D. Benner T. Tseng W. Y. Dai G.. (2008). Diffusion spectrum magnetic resonance imaging (DSI) tractography of crossing fibers. Neuroimage 41, 1267–1277. 10.1016/j.neuroimage.2008.03.036 18495497

[B69] Wisse L. E. Reijmer Y. D. ter Telgte A. Kuijf H. J. Leemans A. Luijten P. R.. (2015). Hippocampal disconnection in early Alzheimer’s disease: a 7 tesla MRI study. J. Alzheimers Dis. 45, 1247–1256. 10.3233/JAD-142994 25697703

[B70] Wu Y. Sun D. Wang Y. Wang Y. Wang Y. (2016). Tracing short connections of the temporo-parieto-occipital region in the human brain using diffusion spectrum imaging and fiber dissection. Brain Res. 1646, 152–159. 10.1016/j.brainres.2016.05.046 27235864

[B71] Yamamoto M. Kushima I. Kimura H. Hayashi A. Kawano N. Aleksic B.. (2015). White matter microstructure between the pre-SMA and the cingulum bundle is related to response conflict in healthy subjects. Brain Behav. 5:e00375. 10.1002/brb3.375 26516610 PMC4614048

[B72] Yeh F. C. Verstynen T. D. Wang Y. Fernandez-Miranda J. C. Tseng W. Y. (2013). Deterministic diffusion fiber tracking improved by quantitative anisotropy. PLoS One 8:e80713. 10.1371/journal.pone.0080713 24348913 PMC3858183

[B73] Yeh F. C. Wedeen V. J. Tseng W. Y. (2010). Generalized q-sampling imaging. IEEE Trans. Med. Imaging 29, 1626–1635. 10.1109/TMI.2010.2045126 20304721

